# The relationship between spiritual health, quality of life, stress, anxiety and depression in working women

**DOI:** 10.3389/fpubh.2024.1366230

**Published:** 2024-08-29

**Authors:** Tayebeh Rakhshani, Pari Saeedi, Seyyed Mansour Kashfi, Leila Bazrafkan, Amirhossein Kamyab, Ali Khani Jeihooni

**Affiliations:** ^1^Department of Public Health, School of Health, Nutrition Research Center, Shiraz University of Medical Sciences, Shiraz, Iran; ^2^Department of Public Health, School of Health, Shiraz University of Medical Sciences, Shiraz, Iran; ^3^Clinical Education Research Center, Shiraz University of Medical Sciences, Shiraz, Iran; ^4^Faculty of Medicine, Fasa University of Medical Sciences, Fasa, Iran

**Keywords:** spiritual health, quality of life, stress, anxiety, depression, working women

## Abstract

**Background:**

While there are benefits to women entering the workforce, there are also drawbacks, such as stress, anxiety, and depression, which can lower quality of life. However, some research indicates that women’s spiritual health may be a protective factor in these situations. This study is to explore the relationship between spiritual health and quality of life, stress, anxiety, and depression among a population of women working in health care centers, given the existence of such a relationship among women.

**Methods:**

In 2022, 500 women who worked in health centers in Izeh City, Iran, participated in this cross-sectional survey. The clustered census sampling approach was used for the sample. The research participants completed a 12-item quality of life questionnaire on spiritual health, stress, anxiety, and depression as part of the data gathering process. The data were examined using independent t tests, one-way variance, and Pearson correlation after being entered into SPSS-24.

**Results:**

Of the participants, 18% were single and 68% were married. In terms of depression, stress, and anxiety, the mean and standard deviation were 8.26 ± 5.78, 11.26 ± 4.89, and 7.91 ± 0.98, respectively. The quality of life had a mean and standard deviation of 30.82 ± 3.56. Women who were unmarried and had more work experience reported a considerably greater quality of life (*p* < 0.05). The findings indicated that stress and spiritual health (*p* = 0.001), anxiety and spiritual health (*p* = 0.032), and depression and spiritual health (*p* = 0.024) all had a significant and inverse association. Furthermore, a strong and positive correlation was found (*p* = 0.001) between spiritual health and quality of life.

**Conclusion:**

The study’s conclusions demonstrated a clear link between spiritual health and life satisfaction, suggesting that working women’s quality of life may be raised by treatments that support spiritual health. Furthermore, the findings demonstrated a significant inverse relationship between spiritual health and the psychological variables of stress, anxiety, and depression. This suggests that by understanding the factors influencing mental health and the role of spiritual health moderator, it may be possible to improve the psychological state of employed women.

## Background

Following the rapid and significant progress of technology, the possibility of social activities in various fields has increased and has caused more people to be attracted to various fields of social activities. The progress of science and technology has caused transformation in all levels of human life, and the type of a new way of life has replaced the traditional way of life (1). This has led to the fact that the need for more labor is no longer met by men, but women should also contribute to turning this cycle around (2). As the most effective members of society, women are actually responsible for the most contribution in managing the family (the most important known social unit). Due to the special role she plays in the creation system, women are equipped with important internal capital that human society needs for its growth (3–5). In the end, women’s employment has had two important consequences: on the one hand, it has led to an increase in the level of women’s education, learning different skills, improving working conditions, women’s tendency to economic independence, and increasing their self-esteem.

On the other hand, working outside the home brings negative consequences such as stress and anxiety, which leads to a decrease in the quality of life for this group (6). The reason for this issue is that women’s work role is a non-traditional role, which mainly causes them to devote more time to their work role due to intense work conflict, and as a result, they face problems doing housework (7). According to the definition of the world organization, an individual’s comprehension of their place in the world within the framework of culture, societal values, and their personal objectives and standards constitutes their quality of life. As a result, it is possible to argue that people’s perspectives on quality of life vary depending on the circumstances and how satisfied they are with their current circumstances. A person’s age, gender, culture, class, education, social environment, and state of health or illness are all related to their quality of life. Ferrell claims that a person’s employment is one of the elements influencing their quality of life (8).

Despite the many positive outcomes associated with women’s increased societal participation, this shift has also introduced specific challenges that primarily affect women, notably in their mental health. Stress and psychological pressure are particularly pronounced in women, who report these issues at higher rates than men. Such stress is often a consequence of the dual burden many women face, juggling long work hours with household responsibilities, which can exacerbate psychological distress (9). This dual role conflict does not just elevate stress but significantly heightens the risk of depression. Depression manifests as a severe and persistent sadness, loss of interest in enjoyable activities, and other debilitating symptoms that disrupt daily functioning. The etiology of depression is complex and multifactorial, encompassing genetic predispositions, neurobiological differences, and psychosocial stressors. Importantly, women experience unique stressors such as gender-based discrimination and societal pressures, which contribute to higher rates of depression compared to men (10). Moreover, research indicates that the societal expectation for women to manage both professional and domestic spheres can lead to chronic stress and mental health declines, making them more susceptible to depression (11). This situation underscores the need for tailored interventions that address these unique pressures and support women’s mental health in the context of modern work and family life.

Depression is a person’s natural response to the pressures of life. In this disorder, the person has states of sadness, cramping, and impatience. The main characteristics of depression are a deep decrease in the desire for everyday enjoyable activities such as socializing, recreation, exercise, and nutrition. Depression is considered to be the only abnormal program that does not fit with the event that caused it (12). Today, unfortunately, depression is one of the most common diseases among people. In depression, the reactions are abnormal; that is, a person becomes too depressed in the face of adversities and failures (13). The causes of this disease can be inheritance, chemical changes, loss of parents in childhood, unpleasant life events, various physical diseases, and the use of drugs. Studies have proven that if someone has experienced great tragedy and sadness during their growth, such as the death of one of their parents or an important family member, they are more susceptible to depression. Depression is one of the most common mental problems among people, so it is estimated that 75% of people admitted to medical institutions suffer from depression (14). The rate of depression among women has increased significantly, so in some research, depression in women is 3–4 times higher than in men, among which housewives have the highest rate of depression (15).

The definition of health according to the World Health Organization (WHO) includes a condition of whole physical, mental, and social well-being in addition to the absence of sickness or infirmity (16). This definition highlights the intricate interplay of various aspects of health, acknowledging that individual well-being extends beyond mere physical conditions to include mental and social factors. However, this definition, while encompassing physical, psychological, and social dimensions, often underestimates spiritual health, which can also significantly impact an individual’s quality of life. Previous research supports the idea that leading a balanced lifestyle, including spiritual well-being, directly contributes to overall life satisfaction (17). Research indicates that leading a healthy lifestyle has an impact on life satisfaction. Physical activity, nutrition, health responsibility, spiritual development, interpersonal relationships, and stress management are the six dimensions of a healthy lifestyle (18, 19). Taking care of one’s spiritual health is one approach to enhance one’s quality of life (20).

One of the crucial facets of human health is spiritual health, which unifies and integrates the relationship between internal forces and is typified by traits like intimacy and contact with God, who has two existential and religious components (21). The fulfillment derived from a relationship with a higher power is the religious dimension, and the pursuit of life’s meaning and purpose is the existential dimension (22). Unlike more stable attributes like physical health, which may fluctuate in response to discrete events (e.g., illness), spiritual health can be more dynamic, influenced by internal and external life changes. Major life events, daily stresses, and even positive developments like career advancement or personal achievements can impact one’s spiritual well-being. When spiritual health weakens, it can lead to a diminished sense of purpose and disconnection, making individuals more vulnerable to mental health issues like depression and anxiety. This vulnerability occurs because a weakened spiritual state may reduce resilience, the ability to cope with everyday stresses and recover from challenges (23). Numerous studies demonstrate the significant influence spirituality and spiritual health have had on raising life expectancy and easing the symptoms of physical and mental illnesses. For instance, Jafari et al.’s study from 2022 examined the impact of the connection between spirituality and health. Bam’s study on spirituality and quality of life in thalassemia patients found that spiritual health had an impact on life satisfaction (24). The findings of a different study by Safara et al. (25), which attempted to connect spiritual health to the psychological coherence of the older adult in Mashreh, demonstrated the direct role that spiritual health plays in averting mental illnesses like anxiety and depression (25).

The relationship between spiritual health and quality of life, stress, anxiety, and depression has been studied in relation to a number of social groups, including cancer patients (26, 27), diabetics (28, 29), the older adult (30–32), and adolescents (33, 34). In recent years, despite the challenges posed by the COVID-19 pandemic, studies continue to highlight the significant impact of spirituality on mental health and overall well-being, underscoring the importance of addressing spiritual health in coping with adversity during times of crisis (35). However, studies have not given as much attention to the group of healthy but working women, despite the fact that they are subjected to disorders like stress, anxiety, and depression, which can ultimately have a significant impact on the quality of individual and marital life. For this reason, the current study aims to investigate the relationship between spiritual health and quality of life, stress, anxiety, and depression among women working in health care centers. Izeh’s city plan and construction were completed.

## Methods

### Research design

A cross-sectional research study was conducted in 2022 involving 500 women employed in health clinics in Izeh City, Iran. The participants were selected based on a series of well-defined inclusion, exclusion, and elimination criteria, designed to ensure the integrity and reliability of the study’s findings.

### Inclusion criteria

Participants were required to be women aged between 25 and 45 years. Both married and unmarried women were eligible to participate, provided they had no history of chronic diseases such as diabetes, hypertension, or cardiovascular conditions. The study also included women regardless of whether they had children, ensuring a diverse representation of parental status. To further refine the sample, only those women who were actively employed at the selected health centers and had no self-reported history of stress, anxiety, or depression being treated were considered eligible.

### Exclusion criteria

Several exclusion criteria were applied to avoid confounding variables that could influence the study’s outcomes. Pregnant women were excluded to eliminate potential bias related to pregnancy-related anxiety or depression. Additionally, women suffering from premenstrual dysphoric disorder (PMDD) were not included, as severe hormonal fluctuations associated with this condition could affect anxiety and depression levels. Lactating women were also excluded to account for postpartum factors that might influence mental health. Furthermore, women undergoing the transition to menopause were excluded due to the significant impact this phase could have on anxiety, depression, and overall quality of life. Lastly, any women who were currently taking antidepressant or anxiety medications at the time of the study were excluded to maintain the study’s focus on untreated psychological conditions.

### Elimination criteria

Elimination criteria were established to manage participant involvement throughout the study. Participants who chose to withdraw their consent at any point were removed from the final analysis. Additionally, any participant who did not complete the required questionnaires or whose responses were deemed inconsistent was excluded from the data set. Moreover, participants found to be violating the study protocol, such as beginning medication during the study, were eliminated to preserve the study’s integrity.

### Sample size and sampling

To determine the sample size by considering the prevalence formula and also according to the results of the study by Jamali Moghadam et al. (34) with the aim of determining the relationship between spiritual health and depression, anxiety, and stress and considering the confidence level of 0.95%, the power of the test was 80% and the dropout rate of 10% was 500 people. After coordinating with the health center in Izeh city and the relevant authorities, the number of health centers and the number of employees in the centers were received. In the first stage, sampling was done as a cluster from all the centers of Izeh city in the four districts of North, South, East, and West. In this way, the centers in these four regions were considered a cluster, and finally, 12 centers were randomly selected from these four regions (three centers in each region). In the second stage, the sampling was done by the census method, and the researcher requested that the staff of the centers participate in the research by referring to these centers, and finally, a total of 500 people participated in the study.

### Ethical considerations

The study was conducted in accordance with the ethical principles outlined in the Declaration of Helsinki. Approval for this research was obtained from the Ethics Committee of Shiraz University of Medical Sciences. The protocol was reviewed and authorized on February 19, 2023, under the authorization code IR.SUMS.SCHEANUT.REC.1402.006. Informed consent was obtained from all participants prior to their involvement in the study. Participants were fully informed about the study’s purpose, procedures, and their right to withdraw at any time without consequence. The confidentiality and anonymity of participants were strictly maintained, with no identifying information being recorded or reported. Participants were assured that their data would be used solely for research purposes and that they could request access to the study’s results if desired.

### Protocol

Before administering the questionnaires, the researchers prepared the necessary materials, including printed copies of the questionnaires and consent forms. The participants have provided with information about the study, its purpose, and what participation involves. Then they were asked to provide their informed consent before participating, and their privacy and confidentiality were ensured throughout the process.

Participants received clear instructions on how to fill out the questionnaires. The questionnaires were completed in a quiet and comfortable environment to minimize distractions and ensure accurate responses. Researchers were available to answer any questions or provide clarifications if participants encounter difficulties understanding certain questions. Once participants have completed the questionnaires, they were gathered by the researchers.

### Data collection instruments

To collect information, a questionnaire containing the personal information of individuals (e.g., age, household size, marital status), the Depression, Anxiety, and Stress Scale 21 (DAS-21), a 12-question quality of life questionnaire, and a spiritual health questionnaire were used.

### Anxiety and depression questionnaire

The DAS-21, or anxiety, stress, and depression questionnaire, was utilized to gather data for this investigation. There are 21 questions on this questionnaire, which are broken down into three subscales: depression, stress, and anxiety. Lovibond et al. (36) designed this tool. People are required to select one of the following four answers for each question: “never,” “a little,” “medium,” or “a lot.” A score ranging from 0 to 3 is presented for each of these possibilities. Each subscale’s score is determined by adding the scores of its questions; by using these scores, one can determine how anxious, stressed, or depressed a person is. Questions 3, 5, 10, 13, 16, 17, and 21 are used to assess depression; questions 1, 4, 7, 9, 15, 19, and 20 are used to measure anxiety; and questions 1, 6, 8, 11, 12, 14, and 18 are used to measure stress.

Each subscale score is calculated by summing the scores from its respective items. The responses for each item range from 0 (never) to 3 (a lot), leading to a potential range of scores that varies depending on the number of items per subscale, and the total score for each subscale is reported. Depression scores are divided into five categories: mild (10–13), moderate (14–20), severe (21–27), and very severe (28). Anxiety scores are categorized as follows: mild (5–8), moderate (10–14), severe (15–19), and extremely severe (20). Determining the normal (0–14), mild (15–18), moderate (19–25), severe (26–33), and very severe (33) stress scores (34). Additionally, according to 34, its reliability coefficients for stress, anxiety, and depression were found to be 0.91, 0.81, and 0.89, respectively. The overall scale coefficient was reported as 0.82, and the reliability coefficients for this scale in Iran were 0.81, 0.78, and 0.80, respectively, in the study conducted by Samani et al. (37).

### Quality of life questionnaire

A condensed version of the 36-question quality of life questionnaire, which is frequently used in research, is the 12-question version. In 1996, Warr, Kasinski, and Keller developed the 12-item quality of life questionnaire. There are eight subscales on this survey. Owing to the limited number of components, the total score of the individual is frequently utilized. The current survey looks at a person’s overall health, physical function, emotional difficulties, physical discomfort, social function, vitality and vital energy, and mental health in order to assess their quality of life. A Cronbach’s alpha of 95% was found when the validity and reliability of the Persian version of the questionnaire assessing the quality of life of impaired individuals were assessed (38).

### Spiritual health questionnaire

The 48-item spiritual health questionnaire was created in Iran and is frequently utilized in research projects. Amiri et al. created the 48-question version of the spiritual health questionnaire in 2015 with the intention of creating and conducting a psychometric analysis of a thorough questionnaire for assessing spiritual health (37). Three conceptual constructs—insight, propensity, and behavior—as well as three sub concepts—communication with God, communication with oneself, and communication with nature—make up this quiz.

The questionnaire comprises three constructs: tendency (7 items for connection with God, 4 items for connection with self, and 5 items for connection with surroundings), behavior (7 items for communication with God, 2 items for communication with self, and 11 items for connection with surroundings), and insight (8 items for connection with God, 1 item for connection with self, and 3 items for connection with surroundings). This questionnaire is scored on a Likert scale of 1 to 5 (numbers typically range from 4 to 5, with the exception of rare instances of 2 and 1), and the scores were calculated as the total number of scores. With the goal of creating and psychoanalytically assessing the spiritual health questionnaire, this questionnaire was assessed in the research of Amiri et al. (39), and it had a 70% Cronbach’s alpha.

### Data analysis

The Kolmogorov–Smirnov test was used to determine the data’s normalcy before SPSS 24 was used to analyze it. The data were analyzed using an independent t-test, one-way variance, and Pearson correlation, and were described using frequency indices, mean, and standard deviation. The Independent *t*-test was used to compare groups such as married vs. unmarried participants. One-way ANOVA was utilized to analyze age groups or years of work experience, which were divided into more than two categories. And the Pearson Correlation was used to evaluate the relationship between variables like spiritual health and quality of life, or anxiety and stress levels. For all tests, the significance threshold was taken to be 0.05, with a 95% confidence interval.

## Results

In this study, 500 women working in the health centers of Izeh participated, with a participation rate of 100%, with an average age of 39.16 ± 3.43. 24% of them were 30–35 years old, 34% were in the age group of 35–40 years, and 42% were in the age group of over 40 years. According to the results, 68% of the participants were married, and 18% of them were single. Table 1 shows the mean and standard deviation of stress, anxiety, and depression variables in the participants. The mean and standard deviation of depression were 8.26 ± 5.78, stress was 11.26 ± 4.89, and anxiety was 7.91 ± 0.98. The mean and standard deviation of the quality of life were 30.82 ± 3.56.

**Table 1:** Frequency distribution of stress, anxiety and depression variables in study participants.

Variable	Normal	Mild	Moderate	Severe	Very severe	Mean	SD
Depression	356 (71.2%)	110 (22%)	26 (5.2%)	5 (1%)	3 (0.6%)	8.26	5.78
Stress	187 (37.4%)	221 (44.2%)	92 (18.4%)	0	0	11.26	4.89
Anxiety	197 (39.4%)	216 (43.2%)	87 (17.4%)	0	0	7.91	0.98
Quality of life						30.82	3.56

Table 2 spiritual health scores in the participants. The mean and standard deviation of spiritual health was 91.54 ± 2.63.

**Table 2:** Distribution of spiritual health scores in the participants.

Variable	Components	Mean	SD
Insight	Connection with God/self	92.31	2.25
	Connection with surroundings	91.45	3.24
	Total score	91.88	2.74
Tendency	Connection with God/self	89.36	1.45
	Connection with surroundings	90.67	3.25
	Total score	90.01	2.35
Behavior	Connection with God/self	92.54	2.21
	Connection with surroundings	91.75	3.22
	Total score	92.14	2.71
Cognitive/emotional component		90.94	2.55
Behavioral component		92.14	2.71
Overall spiritual health		91.54	2.63

The study employed a one-way variance test to investigate the association between marital status, number of children, and employment status. Additionally, an independent *t*-test was employed to ascertain the impact of work experience on quality of life and spiritual health variables. The quality of life did not significantly differ among age groups (*p* = 0.148), the number of children (*p* = 0.650), or employment status (*p* = 0.125), according to the results shown in Table 3. However, there was a significant difference among participants in terms of marital status (*p* = 0.036) and work experience (*p* = 0.020) (Table 3).

**Table 3:** Determining the difference between quality of life according to demographic variables.

Variable		Mean	SD	Test statistics	*p*-value
Age	30–35	36.08	2.35	3325.50	0.148
	35–40	35.73	4.26		
	More than 40	43.28	3.37		
Marital status	Single	45.46	2.25	1.01	0.036
	Married	34.85	5.14		
	Other	34.25	8.67		
Number of children	0–3	37.25	2.33	541.65	0.650
	3–5	34.66	3.71		
	More than 5	36.42	2.64		
Work experience	Less than 6 years	35.73	2.23	0.98	0.020
	More than 6 years	43.28	4.13		

According to the results presented in Table 4, spiritual health according to age groups (*p* = 0.148), marital status (*p* = 0.315), number of children (*p* = 0.128), (*p* = 0.245), and work history (*p* = 0.245) were not significant among the participants (Table 5).

**Table 4:** The relationship between stress, anxiety and depression and quality of life with spiritual health in the participants.

Variables	Spiritual health	
	The significance coefficient	The significance level
Stress	−0.45	0.001
Anxiety	−0.67	0.032
Depression	−0.51	0.024
Quality of life	−0.59	0.001

**Table 5:** Determining the relationship between spiritual health according to demographic variables.

Variable		Mean	SD	*p*-value
Age	30–35	79.08	2.23	0.148
	35–40	80.73	3.26	
	More than 40	79.28	3.44	
Marital status	Single	81.46	4.25	0.315
	Married	82.85	5.26	
	Other	81.25	3.67	
Number of children	0–3	82.25	4.33	0.128
	3–5	83.66	3.27	
	More than 5	83.42	2.64	
Work experience	Less than 6 years	79.73	6.23	0.245
	More than 6 years	78.28	4.13	

The association between stress, anxiety, and depression and spiritual health was examined using Pearson’s correlation test. Table 4 provides a report on the test outcomes. The table indicates a significant correlation (*p* = 0.001) between stress and spiritual health, anxiety, and health. There is a substantial and inverse association between spiritual (*p* = 0.032), depression, and spiritual health (*p* = 0.024). Table 3 shows that there is a significant and direct correlation (*p* = 0.001) between spiritual health and quality of life.

## Discussion

The present study examined the complex relationship between spiritual well-being, quality of life, and psychological discomfort among a sample of 500 employed women at healthcare facilities located in Izeh, Iran. The findings reveal a significant variation in the levels of stress, anxiety, and depression among these women. Significantly, most subjects indicated suffering depression and anxiety at normal to mild levels, while only a minority reported moderate to severe symptoms. The observed distribution could be attributed to the resilience and coping techniques commonly found within this community, which may be influenced by the cultural and social support networks inherent in Iranian society. The results of this study are consistent with previous research conducted by Jamali Moghadam et al. (34), which also found that Iranian women in similar work environments have comparatively low levels of psychological distress.

In addition, our investigation uncovered a subtle connection between age, marital status, employment experience, and the observed psychological results. The quality of life ratings appeared to rise with age, suggesting a cumulative impact of life experiences and ways to build resilience. In contrast, spiritual health scores showed a more consistent distribution across various age groups. This discovery implies that spiritual well-being can maintain a reasonably consistent level across all periods of life, irrespective of environmental influences. The findings are consistent with the observations made by Yousefi Afrashteh et al. (40), who also reported a consistent spiritual well-being status across different age groups in their study on Iranian women.

Our research revealed a notable disparity in quality of life scores between married individuals and singles, with married participants reporting lower scores. This discrepancy could be ascribed to the supplementary obligations and pressures linked to married life. Nevertheless, the ratings for spiritual health did not display a corresponding pattern, indicating that spiritual well-being might act as a safeguard against the adverse effects of marital pressures on overall life satisfaction. The results align with the findings of Javaid et al. (6), who also identified a comparable protective effect of spiritual health against professional stress among married women.

Moreover, the distribution of spiritual health scores among employed women in Izeh exhibited a uniform level across various demographic factors. The consistency seen implies that spiritual well-being is likely to be profoundly rooted in the cultural and theological framework of Iranian society, beyond individual variations in age, marital status, and work experience. The findings are consistent with the earlier study conducted by Amiri et al. (39), which likewise observed consistent spiritual health scores among Iranian communities, irrespective of demographic factors.

Our findings also highlighted a protective role of spiritual health against stress and anxiety, where higher levels of spiritual health correlated with lower stress and anxiety. This protective aspect is critical, echoing the sentiments of Javaid et al. (6) and Yadollahpour et al. (41), who found that stronger spiritual health contributed to lower occupational stress in working women. These results suggest that spirituality might serve as a buffer, moderating the effects of work-related stress through a sense of purpose and connection to a larger existential framework.

Additionally, the relationship between spiritual health and depression presents a compelling insight into the coping mechanisms employed by the participants. In regions with strong religious and cultural ties, such as Izeh, spirituality may play a vital role in managing psychological distress. This is supported by the alignment with Lloyd et al. (42), who found that spiritual practices, such as seeking divine assistance, were inversely related to depression levels.

However, the causal relationships between these variables remain to be elucidated through longitudinal studies. The cross-sectional nature of our study limits the ability to derive causal inferences, thereby necessitating further research to explore these dynamics over time. All in all, this study enriches the existing literature by providing empirical support for the protective effects of spiritual health against psychological distress and enhancing quality of life among working women.

Lastly, this study aligns with previous research highlighting the positive association between spiritual health and quality of life. Our finding is consistent with Yousefi Afrashteh et al. (40) who reported a positive correlation between spiritual health, social support, and life meaning in a sample of Iranian adults. Debnath et al. (43) working with a population in India facing health challenges, and Liu et al. (44) studying chemotherapy patients in China, both documented similar positive influences of spiritual health on quality of life. These findings suggest that spiritual health serves as a significant resource that can bolster quality of life across diverse contexts.

### Strengths and limitations

With a sample size of 500 participants, this investigation could be a great a representative sample from the target population. Additionally, employing established questionnaires for data collection result in enhancing the study’s reliability significantly. This study utilizes various statistical analyses, including Pearson correlation, *t*-tests, and one-way variance tests, to explore the relationships between different variables. This multifaceted approach provides a nuanced understanding of the complex interactions among spiritual health, psychological well-being, and demographic factors.

On the other hand, the study’s cross-sectional design limits its ability to establish causal relationships between variables. Longitudinal or experimental designs could provide stronger evidence for the directionality of associations observed in the study. Moreover, reliance on self-reported measures could result in the potential for response or social desirability bias. Participants may provide answers that they perceive as socially desirable or may not accurately recall their experiences, leading to measurement inaccuracies.

Additionally, our findings may not be generalizable to other demographic groups or cultural contexts. Factors such as cultural norms, socioeconomic status, and access to healthcare may influence the relationships observed in the study. Lastly, while we examined the relationships between spiritual health, psychological well-being, and demographic factors, it might not adequately control for all potential confounding variables. Factors such as socioeconomic status, social support, and coping mechanisms could influence the observed associations but are not fully accounted for in the analysis.

## Conclusion

The study’s findings demonstrated a significant and direct link between spiritual health and life satisfaction, and working women’s quality of life can be raised by interventions that support spiritual health. Furthermore, our findings demonstrated a significant inverse relationship between spiritual health and the psychological variables of stress, anxiety, and depression. Therefore, by understanding the factors influencing mental health and the function of spiritual health moderators among employed women, it is possible to improve psychological state.

### Implications and suggestions

This study has various practical implications. Firstly, since the study highlights the importance of considering spiritual health as a significant determinant of overall well-being, interventions aimed at promoting spiritual health could be integrated into workplace wellness programs, which could potentially reduce stress, anxiety, and depression levels among female employees. Employers and policymakers could collaborate to introduce initiatives such as mindfulness sessions, meditation programs, or access to spiritual counselors within the workplace environment.

Secondly, the findings highlight the need for tailored interventions targeting specific demographic groups. For instance, the study revealed differences in quality of life and spiritual health based on marital status and work experience. Organizations could use this information to develop targeted interventions that address the unique needs of different demographic groups, ultimately fostering a more supportive and inclusive work environment.

Lastly, the research underscores the value of continued investment in longitudinal or experimental studies to further explore the causal relationships between spiritual health, psychological well-being, and demographic factors. By gaining a deeper understanding of these relationships, stakeholders can develop more targeted and effective interventions aimed at improving the overall health and well-being of working women and other demographic groups.

## Data Availability

Data will be made available upon reasonable request from the corresponding author.
